# The British Hospital System, Its Efficiency and Need for Development

**Published:** 1913-07-05

**Authors:** William Osler

**Affiliations:** Regius Professor of Medicine, Oxford.


### THE BRITISH HOSPITAL SYSTEM, ITS EFFICIENCY AND NEED FOR DEVELOPMENT.

By SIR WILLIAM OSLER, Bart., M.D., F.R.C.P., Regius Professor of Medicine, Oxford.

**Being the Presidential Address at the Oxford Hospitals Conference.**

Mr. Vice-Chancellor, Ladies and Gentlemen,—I should like to congratulate this Association on its organisation, on the work which it has done, as an encouragement to the better work which no doubt it will do in the future. To all interested in hospital organisation it must be of the greatest benefit to meet every year and discuss the many problems that come before you. It is exceedingly kind of you to have asked me to be your President this year. I am singularly deficient in the necessary qualifications for such a task. There is probably no one so long and intimately associated with hospitals who really knows less about their administration. Patients, the house physicians, the nurses, and directors I have known intimately, but I have persistently avoided knowing anything about hospital administration as such. If the patients were comfortable, if the beds were clean, if the nurses were happy and the house physicians well housed and well fed, I knew that the administration was sound. It is a pleasure to look back over many years and to feel that I have worked harmoniously with the administration of every hospital with which I have been connected.

There are several points to which I would like to refer. After working for many years in Canadian and American hospitals, it is a very interesting experience to come here and see the contrast that exists. They are many and striking. In Canada the hospitals with which I was associated for many years were organised by Englishmen and by Scotchmen. English rules and English methods were followed. In the United States, when the Johns Hopkins Hospital (with which I was associated for sixteen years) was organised, we followed the German methods of organisation, so that I have been associated with both. Both have advantages and disadvantages into which I cannot here enter. One special feature, I think, in this country is the admirable quality of the smaller hospitals. Go to any county town in England and you will find a hospital, well equipped, clean, and up to date, the patients well looked after—indeed, a thoroughly good, efficient modern hospital; and it is more particularly with reference to the work of these institutions that I wish to speak this morning. In the first place, they arouse an extraordinary amount of interest among the people at large. Take this district, for example; the number of people in the city and in the county who are interested in the Radcliffe Infirmary—prominent citizens, busy men—who are willing to spend much time in its behalf, is very gratifying. Then the management is admirable. The arrangements are excellent so far as the nursing and care of the patients is concerned; and as a rule the operating rooms and dispensary are up to date. The country at large may congratulate itself on having an admirable hospital system. Do not be over anxious that you have fallen upon troublesome days, that you are full of worries as managers—it is good for you, and I hope that your worries may be increased by what I am going to tell you this morning. (Laughter.) It is well to be thoroughly chastened when the rod is upon you!

To four points I wish to refer this morning. The first is the debated one of the voluntary system. My advice is very brief and direct: give it up! It is antiquated, out of date, and it is not going to continue. Make up your minds that you must accept the principle of taking pay from patients. It answers admirably elsewhere. There is not a general hospital in Canada or in the United States which does not take all the money it can get from all classes of patients. There is no difficulty about it. You will have to take it from the insured patients. What reason is there for not taking pay from any patient who can afford it? The practice works well in the Canadian and American hospitals. A patient is asked first if he is a poor man. If so he is taken in without any question. If he is not poor, and comes from within or outside the district, he is admitted at a fixed rate. He is allowed to pay a certain definite amount, or he is asked to pay for operations or special work. There is one striking contrast between the hospitals in this country and those of Canada and the United States. Here you do everything for the poor, who are rich in their hospital care and treatment. Here you do nothing whatever for the poor-rich. (Hear, hear.) They are the most neglected people in the country. If Hodge has acute appendicitis he is taken into a beautiful hospital, operated upon in a splendid operating room, with all the modern advantages; he is put into a big airy ward, and during his convalescence he is out on a balcony, and he has fresh air and is surrounded by all the advantages that hospital administration can give him. What happens to Lady Clara Vere de Vere with acute appendicitis? She is probably taken into a stuffy house transformed into a nursing home; she is operated upon in an upper back room transformed into an operating room. She is transferred to a stuffy room where she stays during her convalescence. No wonder she hates doctors, no wonder she hates nurses and the medical profession. The medical profession is alienated very largely among the upper classes in this country, medical science is alienated, owing to the miserable conditions under which many of the patients of this class have had to live during illness. It would have been a totally different thing if they had been put into beautiful surroundings in many of our general hospitals. (Applause.) There are plenty of nursing homes which are admirable and up to date, but there are a great many that are not; and there is not a nursing home that can take care of a patient as well as a general hospital. (Hear, hear.) What happened in Canada the other day when the

Duchess of Connaught was ill? She was not taken to a nursing home, she was taken to the best place available—to a general hospital. It was her second or her third visit there. I should like to see every general hospital with a big private pavilion. (Hear, hear.) And they pay. There has been a statement current that the private wards in general hospitals do not pay. They do, and they can be made to pay very well.

The second point I would like to worry you about is this. Excellent as are the general hospitals of this country in regard to the care of the patient, the nursing, and the general arrangements—always clean, always tidy, always looking well—when I go to a general hospital I am usually asked to see the wards and the kitchens. I say " No, I do not want to see them, they could not look better here than they did in the last place I visited; show me your clinical laboratory, show me your pathological laboratory." And then the manager has an engagement. (Laughter.) He says " Will you kindly show him that room in the basement? "—(laughter)—and he goes away with a blush that leaves a radiance. And that is what I would emphasise before this Association. You may just as well know the truth, and it is that so far as your clinical and pathological laboratories in the county hospitals are concerned, I will not say they are out of date, because they never were in date, but I say they are shockingly behind the times. You may as well know the truth, and you have got to reform it; you have got to change it. You have got to re-arrange your ideas, because many of you are ignorant on this question. Upon chemical and bacteriological research modern medicine is built, and you cannot have proper treatment of your patients, you cannot have the cases investigated properly, unless you have a good chemical, a good bacteriological, and a good pathological laboratory. In the great majority of the county hospitals those things do not exist, and you should make provision for them at the earliest possible date. They cost money, but that is your business, it is not ours. You are there to provide means for the best possible treatment of the patients, and that you cannot do, without the modern equipment of good laboratories. I hope that at an early date there will not be a single hospital in this country that has to send to the research laboratories in London to have the diagnosis made as to whether a case is one of carcinoma.

The third point is a somewhat delicate one dealing with the medical profession. Medicine is a progressive science, which takes a large share of a man's time if he is working at it thoroughly. I mean what is called internal medicine—the study of disease. In every county in connection with every county hospital there should be a man who is a student working at these problems, and one who is not practising except as a consultant. A hundred years ago nearly every county town in England had its pure physician, and it is a curious piece of history how it is that they should have lapsed, how the conditions have changed, so that now there are very few county towns with hospital physicians who are pure physicians—that is to say, who see cases only in consultation, or who only see purely medical cases. And one reason, I think, is this, that on the medical staff of the hospitals there are often two or three physicians where one would do. It would work much better if a hospital with, say, only fifty or sixty medical beds had but one physician and he a consultant. He should even be paid a fixed stipend sufficient to enable him to devote a very large part of his work to medical investigation and the care of the patients. With the development of modern medicine it takes so much of a man's time for special study and research that it is impossible, if he has to earn his living by taking care of patients in general practice, to keep up to date thoroughly in laboratory methods and in methods of research. In hospitals with a hundred and fifty or two hundred beds the managers should look forward to the day when one pure physician had charge of the medical department instead of, as at present, three or four men in general practice.

The fourth point I would bring before you is the importance of utilising the county hospitals for purposes of instruction. In the old days they were utilised by the medical students and by the apprentices, and every county hospital had five or six or sometimes a dozen medical students in attendance during the Long Vacation. Now it is the rarest possible thing to see a medical student. There is a work they can do which is of the greatest importance—the county hospital should be not only the consulting centre for the doctors of the neighbourhood, but it should be the centre to which they should come for systematic instruction. No body of men need more persistent brain-dusting than do doctors. The profession of medicine is progressing at such a rate that in five or six years a man's knowledge is rusty, and it is a most important thing for the public that the average doctor should keep up to date. One way in which he can keep himself thoroughly informed on the progress of knowledge is by having post-graduate courses in connection with the county hospital. I hope that a British committee which has this post-graduate instruction in hand will present within this year to each county hospital a scheme dealing with this matter. It can be very easily and satisfactorily arranged. The idea is to have, say in the month of October, once a year, possibly in some places twice a year, a week devoted to post-graduate teaching which would be conducted partly by the staff of the hospital, partly by men who would be invited to come, and partly by a group of men who may be under the control of this committee for post-graduate instruction; that is to say, certain specialists throughout the country will be ready to come here, for instance, for four or five days and give instruction or be ready to go to Worcester, or Gloucester, or Norwich, or anywhere they were called upon. I hope next autumn we shall have a post-graduate week here at the Radcliffe Infirmary; and I am sure that throughout the country at large it would be not only a means of conveying a great deal of valuable information to the doctors in each community, but it would help to link the doctors more closely with the hospital, and be a mutual benefit. The plan has worked admirably on the Continent, and I think it should be introduced here. It is not expensive, and the cost could be very readily met by the profession of the country.

These are the four points I wanted to bring before you. They are not, perhaps, connected directly with hospital organisation, but they are connected very intimately with the work which you have to control. And the two points I would particularly impress upon you are the necessity for more scientific work in the way of clinical laboratories and pathological investigation, and the necessity of making every hospital over which you have control a centre for the whole profession. No doubt the linking of the hospital in the Insurance scheme will promote that very much, for you should make every panel doctor feel that the hospital is the place to which he may go for advice and for aid in his special emergencies. If you alienate the panel doctor from the general hospital you have only one alternative in this country, and that is State service; you have got to work your hospital with the panel system, or else the panel authorities will have their own municipal scheme, and you will have rival institutions.

I thank you for having listened to me so patiently.

